# Pulmonary Alveolar Proteinosis in Setting of Inhaled Toxin Exposure and Chronic Substance Abuse

**DOI:** 10.1155/2018/5202173

**Published:** 2018-01-23

**Authors:** Meirui Li, Salem Alowami, Miranda Schell, Clive Davis, Asghar Naqvi

**Affiliations:** ^1^Michael G. DeGroote School of Medicine, McMaster University, Hamilton, ON, Canada; ^2^Department of Pathology and Molecular Medicine, McMaster University, Hamilton, ON, Canada; ^3^Divisions of Critical Care, Respirology, and General Internal Medicine, Department of Medicine, McMaster University, Hamilton, ON, Canada

## Abstract

Pulmonary alveolar proteinosis (PAP) is a rare lung disorder in which defects in alveolar macrophage maturation or function lead to the accumulation of proteinaceous surfactant in alveolar space, resulting in impaired gas exchange and hypoxemia. PAP is categorized into three types: hereditary, autoimmune, and secondary. We report a case of secondary PAP in a 47-year-old man, whose risk factors include occupational exposure to inhaled toxins, especially aluminum dust, the use of anabolic steroids, and alcohol abuse, which in mice leads to alveolar macrophage dysfunction through a zinc-dependent mechanism that inhibits granulocyte macrophage-colony stimulating factor (GM-CSF) receptor signalling. Although the rarity and vague clinical presentation of PAP can pose diagnostic challenges, clinician awareness of PAP risk factors may facilitate the diagnostic process and lead to more prompt treatment.

## 1. Introduction

Pulmonary alveolar proteinosis (PAP) is a rare lung disease characterized by the accumulation of proteinaceous material in the alveoli due to decreased clearance of surfactant by alveolar macrophages. First described in 1958, the prevalence of PAP has been estimated to be 6.87 per million [1, 2].

PAP patients can present asymptomatically or with vague complaints of dyspnea and cough [3]. High resolution computed tomography (HRCT) scan of the lungs show either bilateral diffuse ground-glass opacity or the characteristic “crazy-paving” pattern [4]. Bronchoalveolar lavage (BAL) is used in diagnosis in up to 83% of cases in recent years, replacing lung biopsy as the diagnostic tool of choice [5, 6].

There are three types of PAP: hereditary, autoimmune, and secondary. Hereditary PAP mainly affects children, while the latter two occur in adults [3]. Here, we report the case of a patient who developed PAP secondary to environmental exposure in the setting of chronic alcohol and steroid abuse.

## 2. Case Report

A previously well 47-year-old man presented with progressive dyspnea on exertion and fleeting bouts of sharp precordial chest pain for several months. Echocardiogram showed mild diastolic dysfunction and wall motion abnormalities on Persantine Sestamibi stress test, leading to the diagnosis of heart failure. However, the patient's symptoms worsened in the subsequent months despite being on optimal management for heart failure and he was admitted to the hospital. An in-patient cardiac catheterization showed no significant obstruction in the coronary arteries, prompting investigations for a pulmonary etiology.

The patient was a lifelong nonsmoker who worked as an elevator mechanic with occasional exposure to small quantities of hazardous materials such as aluminum dust, asbestos, paint fumes, hydraulic oil, and cleaning agents. There is no increase in the exposure immediately prior to the onset of dyspnea. He reported a history of chronic alcohol consumption of 6–12 beers per day, as well as regular use of anabolic steroids. He had no past history of hematological cancers, chemotherapy, or radiation.

**Figure 1:**
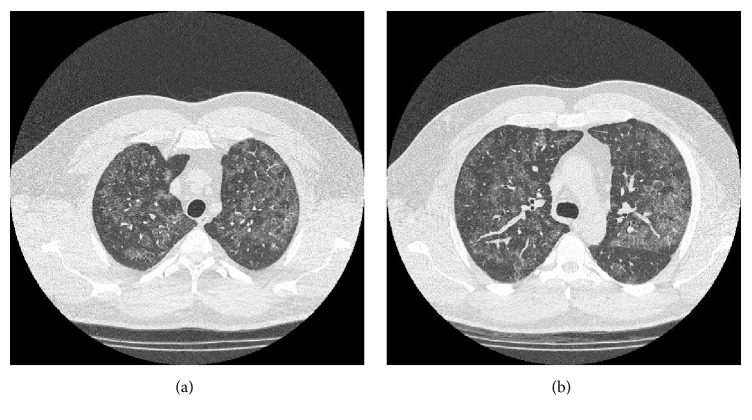
(a) and (b) Bilateral diffuse ground-glass opacities with thickened interlobular septae were seen in multiple sections of the lungs on HRCT.

Physical exam revealed a heavily muscled man of 270 lbs with abdominal obesity. There were no audible wheezes or signs suggesting consolidation on auscultation of the lungs. Cardiac exam revealed normal heart sounds, absence of murmurs or rubs, and a nondisplaced apex. Arterial blood gas analysis revealed an alveolar-arterial gradient of 15 mmHg, consistent with gas transfer defect. Except for a mild diffusion defect (DLCO 81% of predicted), spirometry was normal with a FEV1 of 4.52 (110% of predicted) and FVC of 5.5 (103% of predicted), and a FEV1/FVC ratio of 81%. A 6-minute walk test was sufficient to cause dyspnea and a drop in oxygen saturation from 94% to 91%. Diffuse airspace disease was noted on chest X-ray. An HRCT scan demonstrated bilateral diffusely scattered zones of ground-glass opacification with accentuation of the interlobular septae, suggesting alveolar disease. This combination of findings characterizes the so-called “crazy-paving pattern” commonly seen in PAP. There is no convincing evidence of interstitial lung disease, nor any signs of air trapping that would suggest an obstructive etiology (Figure 1).

**Figure 2:**
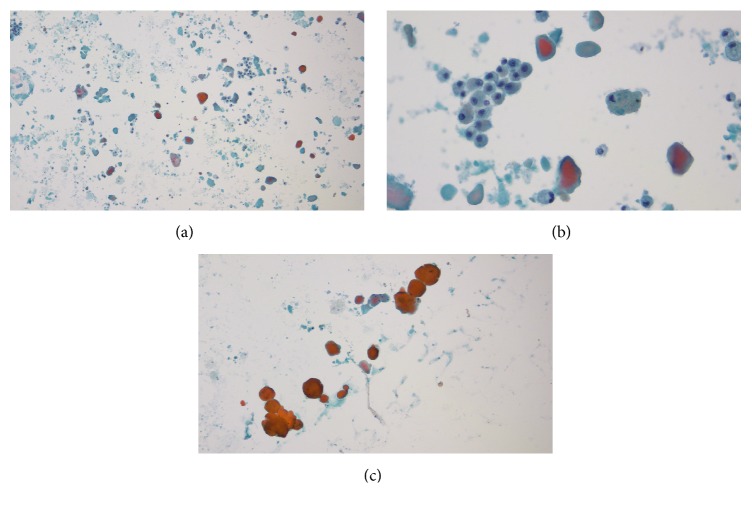
(a) Papanicolaou-stained smear of BAL fluid (lower magnification). (b) Papanicolaou-stained smear of BAL fluid (higher magnification) showing amorphous globules and alveolar histiocytes (likely macrophages) with dense intracellular proteinaceous material. (c) Paucicellular, amorphous globules staining orange, green, and green with orange centre under Papanicolaou-stain (higher magnification).

**Figure 3:**
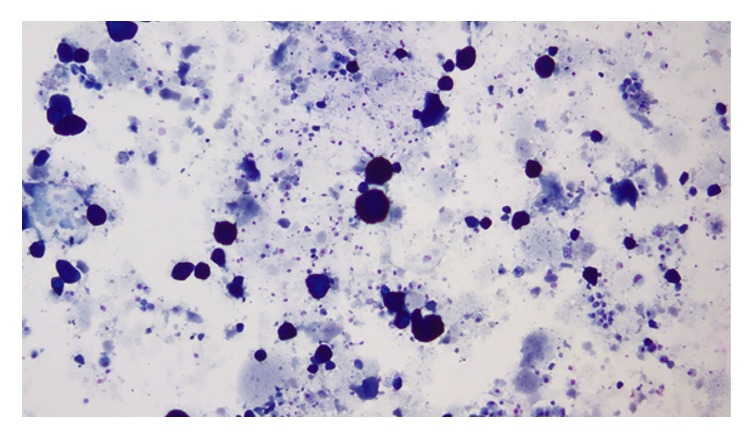
Diff-Quick stain of BAL fluid showing densely blue, basophilic globules.

**Figure 4:**
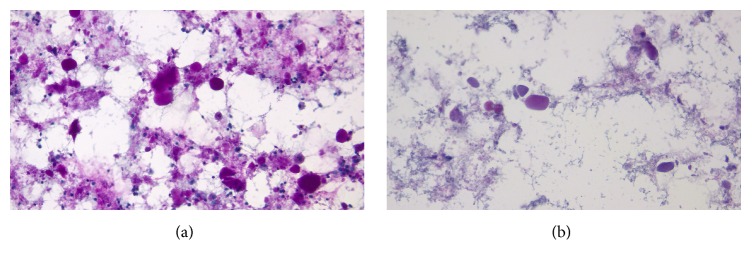
(a) PAS stain of BAL fluid. Globules were PAS-positive. (b) The globules were diastase-resistant.

**Figure 5:**
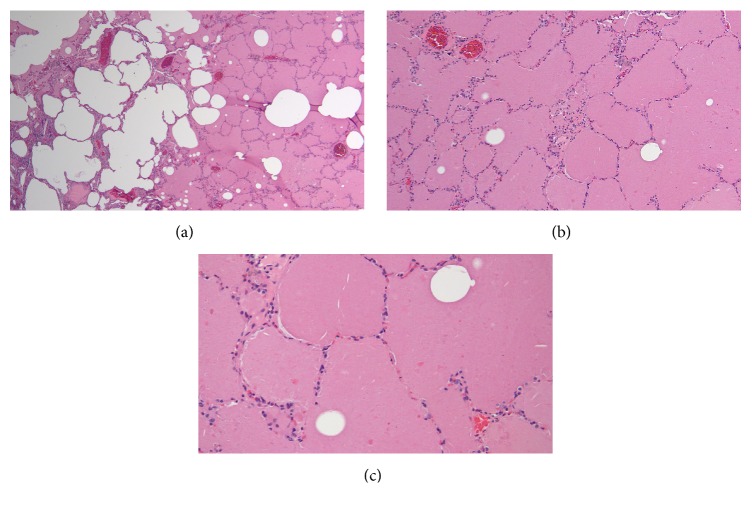
H&E stain of lung wedge biopsy sections at 100x (a), 200x (b), and 400x (c) magnifications showing intact alveolar walls, normal interstitium, and amorphous, eosinophilic granular material in the airspace.

BAL of the lungs yielded milky and opalescent fluid. Amorphous green, orange, and orange-centred globules and histiocytes containing dense proteinaceous material were seen on Papanicolaou-stained smears of BAL fluid (Figure 2). These globules appeared densely dark blue and basophilic under Diff-Quick stain and were periodic acid-Schiff- (PAS-) positive and diastase-resistant (Figures 3 and 4), consistent with the diagnosis of PAP. Blood sample was tested for antibodies against granulocyte macrophage-colony stimulating factor (GM-CSF) through an outside lab and was reported negative. As standard treatment, the patient was set to undergo whole-lung lavage (WLL). When initial washings did not yield significant amount of secretions, the diagnosis of PAP was questioned and WLL was converted to wedge biopsy of the lung in the operating room. H&E stains of the wedge biopsy showed intact alveolar structure filled with amorphous granular eosinophilic material and no interstitial pathology, confirming the diagnosis of PAP (Figure 5). Following the result of the biopsy, a second WLL was scheduled for the patient. Pulmonary testing one year later showed that DLCO had improved from 81% to 98% of predicted; FEV1, FVC, and VC were again normal. On exercise testing, the patient was able to achieve a low-normal work capacity of 80% of maximum predicted value, with normal oxygen saturation at rest and on exertion.

## 3. Discussion

Autoimmune and secondary PAP are the two acquired forms of PAP affecting adults. Autoimmune PAP, which makes up 90% of reported PAP cases [3], is caused by the production of autoantibodies against GM-CSF, a crucial cytokine in the maturation of monocytes into macrophages [7]. Secondary PAP occurs in the setting of macrophage dysfunction caused by infections, hematological disorders, immunosuppression, and exposure to hazardous materials [3]. Hematologic disorders are the most common etiology for secondary PAP, accounting for an estimated 88% of cases [8]. The exact incidence of PAP due to inhalational exposure is unknown. Secondary PAP patients have negative serology for anti-GM-CSF antibodies [3]. The standard therapy for PAP is whole-lung lavage. Although exogenous GM-CSF has been shown to be safe and beneficial for autoimmune PAP, it is not routinely used for secondary PAP [9].

Our patient reported exposure to inhaled toxins such as aluminum dust, organic dust comprised of human sheddings, and fumes of paint and cleaning products. Inhaled dusts and fumes have been described as risk factors for secondary PAP in studies and case reports. Silica dust is the most frequently reported hazardous substance (21% in a single-centre German study), followed by aluminum dust (18%). Exposure to fumes of paint and cleaning products have been reported by 13% and 8% of patients, respectively [6].

Unlike autoimmune PAP, macrophage dysfunction in secondary PAP occurs through a mechanism that is largely independent of anti-GM-CSF antibodies. This is evident in the case of a Japanese patient who developed secondary PAP two weeks after being exposed to silica and aluminum dust particles following the Great East Japan Earthquake. The patient's serum was observed to have an inhibitory effect on GM-CSF signalling despite the paucity of anti-GM-CSF antibodies [10].

Ongoing controversy exists regarding the association between inhalational exposure and the development of autoimmune PAP. It has been reported that exposure to indium compounds, which are used as transparent conductive coating on flat-screen electronic displays, can lead to the production of autoantibodies against GM-GSF [11]. The percentage of autoimmune PAP patients who also had inhalational exposure varies between 26% and 54% in studies [6, 12, 13]. In one Chinese study, this percentage is not significantly different from the prevalence of inhalational exposure in non-PAP hospital controls [13]. While it is possible that exposure to inhaled toxins plays a role in the pathogenesis of autoimmune PAP, stronger evidence is needed.

Our patient has a history of alcohol and steroid abuse concomitant to occupational exposure of inhaled hazardous substances. Although there are no previous reports linking alcohol abuse to PAP, alcohol has been demonstrated to inhibit alveolar macrophage function in animal and molecular models. Mice subjected to chronic ethanol ingestion are shown to have suppressed cytokine secretion and macrophage phagocytosis in the alveoli. Ingesting ethanol for six weeks leads to decreased GM-CSF receptor expression on murine alveolar macrophages and impaired binding of transcription factor PU.1 to the receptor [14]. The effect of alcohol on GM-CSF signalling may be zinc-dependent. Zinc is an important cofactor in cellular processes including GM-CSF signalling. Ethanol ingestion in mice leads to decreased expression of zinc transporters on murine alveolar macrophages and increased risk of zinc deficiency through malnutrition [15]. Both GM-CSF therapy and zinc supplementation are able to restore macrophage phagocytic function in ethanol-fed mice, further supporting the link between alcohol and alveolar macrophage dysfunction [15, 16]. Similarly, corticosteroids have not been established as a causative agent of secondary PAP. Corticosteroids are routinely used to treat neonatal respiratory distress syndrome by increasing Type II alveolar cell surfactant production [17]. Macrophage function may also be impaired through the immunosuppressive effect of systemic corticosteroids. A retrospective cohort study done in 2015 found that corticosteroid therapy in autoimmune PAP patients lead to higher disease severity score in a dose dependent manner. Furthermore, the authors concluded that the worsening of disease is likely due to corticosteroids exacerbating the pathogenic process of PAP per se and cannot simply be attributed to the increased rates of opportunistic infections secondary to immunosuppression [18]. Therefore, it is possible that steroid abuse also had a synergistic effect on the patient developing PAP.

Although hereditary PAP has been described as a disease affecting children and infants, since 2010 three cases of adult-onset hereditary PAP have been reported in literature. All three patients tested negative for anti-GM-CSF antibodies and had elevated serum GM-CSF levels and mutations in either CSF2RA or CSF2RB gene encoding parts of the GM-CSF receptor. Serum GM-CSF level and genetic testing have not been done on our patient [19–21]. Although it cannot be ruled out, given the extreme rarity of adult-onset hereditary PAP and the presence of risk factors such as inhalational exposure, secondary PAP is a much more likely etiology. Long-term follow-up is needed to determine whether cessation of exposure prevents future recurrences of PAP.

## 4. Conclusion

Inhalational exposure to toxic dusts and fumes is a major risk factor for developing secondary PAP. Although a link between alcohol abuse and PAP has not been established, ethanol ingestion has been demonstrated to suppress GM-CSF signalling and macrophage function in mice models. It is possible that alcohol and steroid abuse have played contributory roles in the pathogenesis of PAP in this patient. The rarity and vague clinical presentation of PAP can pose diagnostic challenges. The presence of medical and social risk factors of PAP should raise suspicion for PAP in patients presenting with respiratory symptoms with no clear cause. Increasing clinician awareness for these risk factors can shorten the time to diagnosis and treatment, potentially improving patient outcome.
